# Influence of Zirconium Addition on Heat Treatment Response, Microstructure, and Corrosion Resistance of AA7075 Alloy for Aerospace Applications

**DOI:** 10.3390/ma19214712

**Published:** 2026-11-05

**Authors:** Badegül Lale

**Affiliations:** Aircraft Engineering Department, Faculty of Aviation and Space Sciences, Sivas University of Science and Technology, 58000 Sivas, Turkey; btuncay@sivas.edu.tr

**Keywords:** AA7075, Zirconium, powder metallurgy, RRA heat treatment, microstructure, corrosion

## Abstract

The AA7075 alloy is widely used in the aerospace industry, particularly for its high strength-to-weight ratio in the production of critical structural components such as wing spars, fuselage frames, landing gear supports, rocket booster casings, and high-stress fasteners. This study was designed to enhance the strength and service performance of the AA7075 alloy. In this study, AA7075 alloy was mechanically alloyed with different amounts of Zr (0, 0.075, 0.15, and 0.30 wt.%), followed by cold pressing and sintering in a controlled atmosphere furnace. The AA7075 + Zr alloys produced were subjected to retrogression and re-aging (RRA) heat treatment. The microstructural characteristics of the RRA-treated AA7075 + Zr alloys were characterized using scanning electron microscopy (SEM) and X-ray diffraction (XRD). The mechanical properties were evaluated through density, microhardness, and corrosion tests. Consequently, the influence of different Zr additions on the RRA heat treatment response of AA7075 alloy produced by (MA) Mechanical alloying was investigated. Microstructural examinations, including SEM imaging and EDS analysis, revealed that Al was the predominant element, while variations in elemental composition were observed in certain regions. According to the XRD results, Al, Al_2_CuMg, Al_2_Cu, and MgZn_2_ phases were identified. With the addition of Zr, Al_3_Zr, Al_3_Zr_4_, and Al_3_Zr_5_ phases were formed. The lowest surface roughness (Ra) value of 0.078 µm was obtained for the AA7075 + 0.30 wt.% Zr alloy. The corrosion tests conducted in a 3.5% NaCl solution demonstrated a progressive improvement in the corrosion resistance of the AA7075 alloy as the Zr content increased. The corrosion rate was 1.9 mm/year for the AA7075 alloy, decreasing to 0.19 mm/year for the AA7075 + 0.30 wt.% Zr alloy. SEM examination of the corroded surfaces revealed localized surface degradation characterized by the formation of corrosion pits, indicating a predominantly pitting corrosion mechanism.

## 1. Introduction

Aluminum alloys have been extensively used in recent years, particularly in the aerospace industry, owing to their superior mechanical properties, advanced manufacturing processes, design flexibility, and ease of inspection. Due to their favorable combination of high strength, low density, fatigue resistance, fracture toughness, and corrosion resistance, these alloys have been extensively investigated and further developed in both industrial and academic research [1,2]. Among the high-strength aluminum alloys used in the aerospace industry, AA7075 (Al-Zn-Mg) has an important place and has been widely investigated in the literature. AA7075 alloy contains approximately 5.6% Zn, 2.5% Mg, and 1.6% Cu. The Mg and Zn present in the alloy play an important role in the precipitation-hardening mechanism, and the precipitates formed during heat treatment enhance the mechanical properties of AA7075 [3].

AA7075 alloy can be produced conventionally by casting, forging, extrusion, and semi-solid forming. In recent years, powder metallurgy (PM) has been considered a promising alternative to these conventional manufacturing methods due to its capability to produce components with complex geometries in near-net-shape form. In powder metallurgy production, reduced material waste and lower requirements for subsequent machining provide significant advantages, particularly for small and complex-shaped components. In addition, alloying elements can be added to the powder mixture in desired proportions, allowing better control of the chemical composition and facilitating the development of different alloy compositions [3]. Owing to these advantages, PM is considered a suitable manufacturing route for AA7075-based aerospace and automotive components that require high specific strength and low weight [4]. The properties of AA7075 alloy depend not only on its chemical composition but also on the heat treatment conditions applied. T6 is one of the most commonly used heat treatments for AA7075. In this treatment, the alloy is first solution-treated and quenched, then artificially aged. During aging, fine η′-MgZn_2_ precipitates form within the Al matrix. These precipitates hinder dislocation movement and thereby increase the hardness and strength of the alloy. However, precipitates formed along the grain boundaries after T6 treatment, together with the precipitate-free zones adjacent to these regions, may adversely affect corrosion behavior. Therefore, AA7075 alloy in the T6 condition may exhibit increased susceptibility to intergranular corrosion and stress corrosion cracking.

For this reason, retrogression and re-aging (RRA) treatment can be applied after T6 heat treatment. During the retrogression stage, the alloy is exposed to a higher temperature for a short period, promoting the coarsening of grain boundary precipitates and leading to a more discontinuous and uniform distribution. Subsequent re-aging promotes the re-formation of fine precipitates within the grains and helps recover most of the hardness lost during retrogression. Thus, RRA treatment aims to improve corrosion resistance while maintaining the high strength achieved in the T6 condition [5,6,7]. In addition to heat treatment, alloying elements are also used to obtain the desired properties in AA7075 alloy. Zr is one of these elements and promotes the formation of Al_3_Zr dispersoids in the Al matrix. The fine structure of these dispersoids and their stability at high temperatures are important to the alloy’s microstructure. Al_3_Zr dispersoids restrict the movement of dislocations and subgrain boundaries, thereby limiting recrystallization and grain growth. As a result, they contribute to maintaining the grain structure, particularly at elevated temperatures, and improve the mechanical properties of AA7075 alloy [8,9].

The changes resulting from Zr addition are related not only to the amount of Zr added to the alloy but also to the size and distribution of Al_3_Zr particles formed in the microstructure. As the Zr content increases, the formation of coarse primary phases may limit the expected contribution of Zr. Therefore, determining an appropriate Zr content is important. In this study, the effects of different Zr additions on the microstructure, hardness, and corrosion behavior of AA7075 alloy after T6–RRA heat treatment were investigated. In aerospace applications, AA7075 alloys may be subjected to repeated loads, corrosive environments, and thermal stresses from temperature fluctuations during service. These conditions can lead to fatigue damage over time. In particular, repeated loading plays an important role in the initiation and propagation of fatigue cracks [10,11].

Abonama et al. [12] investigated the effect of Ti addition on the corrosion behavior of AA7075 alloy. Ti was added to AA7075 at 2, 4, and 8 wt.% using an Al–Ti master alloy through a casting process. The produced AA7075–Ti alloys were subjected to T6 aging heat treatment and 6% cold rolling, and their corrosion behavior was examined in a 3.5% NaCl solution. The corrosion resistance of the alloys was evaluated using immersion and potentiodynamic polarization tests. The results showed that both Ti addition and cold rolling improved the corrosion resistance of the alloys. However, in the Ti-free AA7075 alloy, cold rolling increased precipitate formation, resulting in decreased corrosion resistance. Martin et al. [3] investigated the effect of Mg on the sintering behavior, microstructure, and mechanical properties of Al–Zn–Mg–Cu alloys with a chemical composition similar to that of AA7075. In their study, 0–4.5 wt.% Mg powder was added to a pre-alloyed Al–5.5Zn–2.5Mg–1.6Cu powder, and the resulting Al–5.5Zn–2.5Mg–1.6Cu + 0–4.5 wt.% Mg alloys were subjected to T6 heat treatment. The results showed that increasing the Mg content reduced the optimum sintering temperature while increasing the required sintering time. An increase in the total Mg content up to 3.5 wt.% was reported to be beneficial in terms of sintering behavior and mechanical properties. However, at higher Mg contents, the expected improvement was limited because the secondary phases formed in the microstructure could not be completely dissolved during solution treatment. The authors further noted that the mechanical properties were adversely affected by the presence of large pores and lubricant residues, as well as by the accumulation of Fe-containing intermetallic phases along the grain boundaries.

Zhang et al. [13] investigated in detail the effect of different Cu additions on the mechanical properties and microstructural evolution of an Al–Mg–Zn (Cu) crossover alloy. The addition of Cu to the newly developed Al–Mg–Zn alloy increased the yield strength by 80.5% without causing a significant loss in ductility. Cu addition contributed to both solid-solution and precipitation strengthening and accelerated the aging response, demonstrating its effectiveness in achieving high strength in Al–Mg–Zn-based alloys.

The corrosion resistance of alloys produced by PM varies depending on the mechanical alloying time and the chemical composition of the alloy. During mechanical milling or alloying, repeated fracture and cold welding alter the size and morphology of the powder particles, which in turn affect the mechanical properties and corrosion behavior of the material [14]. However, the formation of a certain amount of porosity is unavoidable in materials produced by PM. The channels formed by interconnected pores facilitate penetration of the corrosive solution into the material’s interior, thereby increasing its susceptibility to electrochemical corrosion [15].

Numerous studies have investigated the addition of various alloying elements to AA7075 and the application of different heat treatments. However, studies jointly examining the effects of RRA on the microstructure, mechanical properties, and corrosion behavior of Zr-modified AA7075 alloys produced by PM remain limited. In particular, determining how secondary phases form via Zr addition and how precipitates change during RRA affect corrosion behavior, which is essential for assessing the reliability of these alloys in aerospace applications. In this study, AA7075 alloys containing different Zr contents were produced by PM and subsequently subjected to RRA heat treatment. This study addresses this gap in the literature by investigating the effects of Zr content and RRA heat treatment on the microstructure, hardness, and electrochemical corrosion behavior of AA7075 alloys.

## 2. Materials and Methods

The chemical compositions of the pre-alloyed AA7075 powder (115 μm) and the Zr powder (99.9% purity, 149 μm) used in this study are presented in Table 1. The powders were supplied by Nanografi (Ankara, Türkiye).

**Figure 1:**
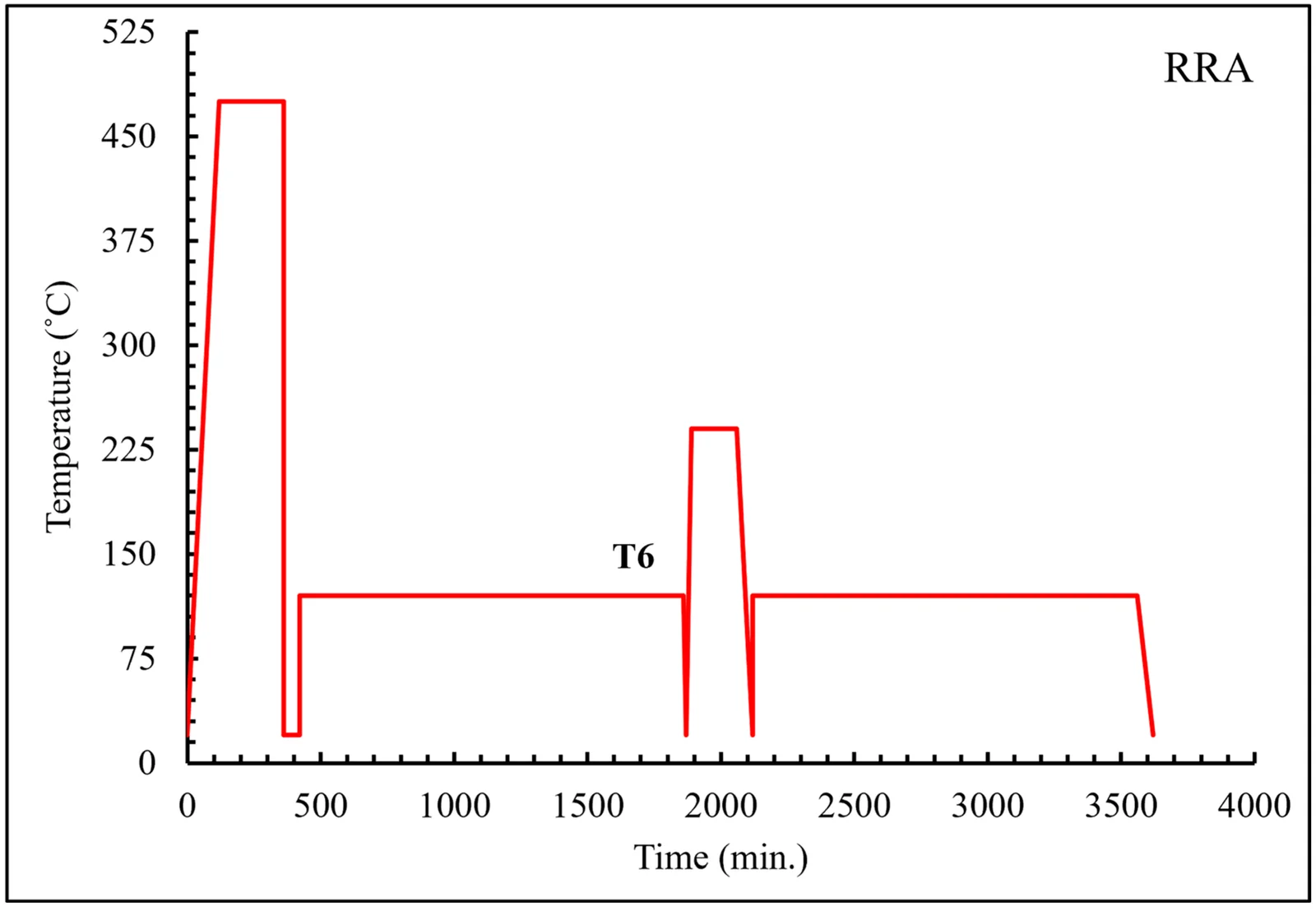
RRA heat treatment applied to AA7075 and AA7075 + Zr alloys.

Pre-alloyed AA7075 powders were mechanically alloyed with 0, 0.075, 0.15, and 0.30 wt.% Zr in a planetary ball mill (FRITSCH GmbH, Idar-Oberstein, Germany) at 400 rpm for 150 min, using 10 mm stainless steel balls and a powder-to-ball mass ratio of 1:10. Methanol (2%) was added as a process control agent. The resulting AA7075 + Zr powders were uniaxially compacted at 550 MPa in a die to produce green compacts approximately 10 mm in diameter and 10 mm in height. The green AA7075 + Zr compacts were sintered in an atmosphere-controlled furnace at 600 °C for 1 h under a vacuum. The sintered alloys were subsequently subjected to RRA heat treatment. A schematic representation of the RRA heat treatment applied to AA7075 and AA7075 + Zr alloys is shown in Figure 1.

Following RRA heat treatment, the AA7075 and AA7075 + Zr alloys were prepared for microstructural examination using standard metallographic procedures in accordance with ASTM E3-11 [16] and etched with Keller’s reagent for 30–40 s. The microstructures of the fabricated AA7075 and AA7075 + Zr alloys were examined using a field-emission scanning electron microscope (Ultra Plus Gemini, Carl Zeiss Microscopy GmbH, Oberkochen, Germany), and X-ray diffraction (XRD) analyses were performed at room temperature using a Rigaku Ultima IV diffractometer (Rigaku Corporation, Tokyo, Japan) equipped with a copper X-ray tube and Cu Kα radiation. Density measurements were conducted using a Precisa density measurement kit (Precisa Gravimetrics AG, Dietikon, Switzerland), while Vickers microhardness tests were performed using a Shimadzu HMV microhardness tester (Shimadzu Corporation, Kyoto, Japan) under a load of 10 N with a dwell time of 10–15 s. Five microhardness measurements were taken for each fabricated AA7075 and AA7075 + Zr alloy, and the results were evaluated based on their arithmetic mean. Surface roughness measurements were performed using a Mitutoyo Surfest S5-210 instrument (Mitutoyo Corporation, Kawasaki, Japan) at a scanning speed of 0.5 mm/s.

Corrosion measurements were carried out by potentiodynamic polarization tests in a 3.5% NaCl aqueous solution. The fabricated AA7075 and AA7075 + Zr alloys were immersed for 3600 s to allow the open-circuit potential (OCP) to stabilize. Polarization measurements were conducted using a three-electrode configuration, with Ag/AgCl as the reference electrode and graphite as the counter electrode (CE). Cyclic polarization measurements were performed over a potential range of −1.0 to +1.5 V at a scan rate of 1.5 mV/s. A fresh solution was used for each test. The corrosion potential (E_corr_) and corrosion current density (j_corr_) were obtained from the polarization data. The corrosion rate was calculated from j_corr_ according to ASTM G102 [17] using the following equation:

(1) $$CR=0.00327\;\frac{j_{corr}\times EW}{\rho }$$

where CR is the corrosion rate (mm/year), j_corr_ is the corrosion current density (µA/cm^2^), EW is the equivalent weight, and ρ is the density of the alloy (g/cm^3^). After the corrosion tests, the surfaces of the AA7075 and AA7075 + Zr alloys were examined by SEM.

## 3. Results and Discussion

### 3.1. Microstructural Characterization

**Figure 2:**
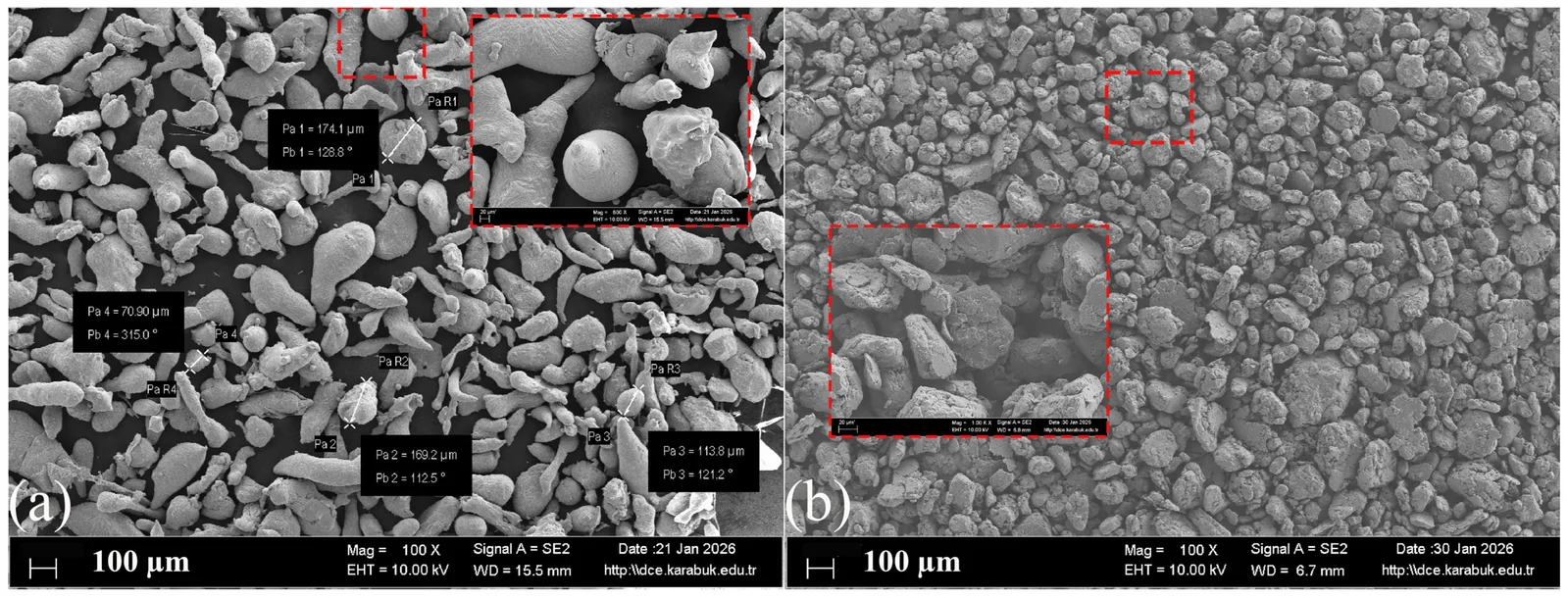
SEM images of the powder particles used in this study: (**a**) pre-alloyed AA7075 and (**b**) Zr.

SEM images of the pre-alloyed AA7075 and Zr powder particles used in this study are presented in Figure 2.

**Figure 3:**
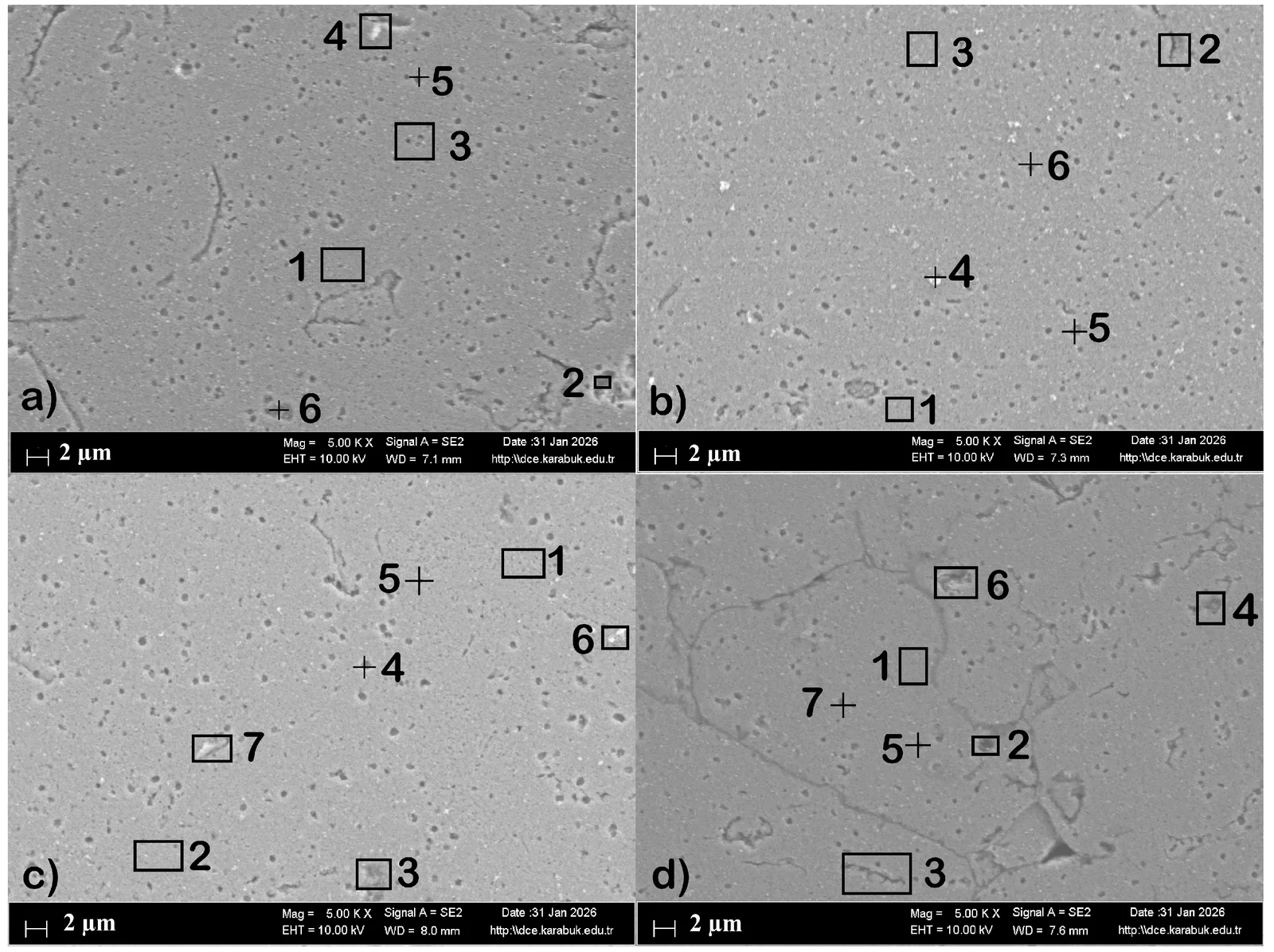
SEM images after RRA treatment: (**a**) 0.0 wt.% Zr, (**b**) 0.075 wt.% Zr, (**c**) 0.15 wt.% Zr, and (**d**) 0.30 wt.% Zr.

SEM images of the pre-alloyed AA7075 and Zr powder particles used in this study are presented in Figure 2. The red dashed boxes denote the selected regions, which are presented at higher magnification to offer a more comprehensive view of the powder morphology. The pre-alloyed AA7075 powder particles (Figure 2a) exhibit predominantly oval or rod-like morphologies, whereas the Zr powder particles (Figure 2b) display angular, irregular, flattened, and flake-like morphologies. The surfaces of the pre-alloyed AA7075 powder particles are also smoother than those of the Zr particles. Figure 3 presents SEM images of the AA7075 + Zr alloys containing 0.0, 0.075, 0.15, and 0.30 wt.% Zr after MM, cold compaction, sintering, and RRA heat treatment.

Figure 3 shows the microstructures of the AA7075 alloy and alloys containing different Zr contents after RRA heat treatment. In general, fine precipitates dispersed throughout the matrix, along with occasional pores and grain boundaries, are observed in all samples. In the AA7075 alloy without Zr addition (Figure 3a), the grain boundaries are distinct, while the precipitates within the matrix exhibit a relatively nonuniform and sparse distribution.

With the addition of 0.075 wt.% Zr (Figure 3b), the number of fine particles within the Al matrix increased, and the precipitates exhibited a more homogeneous distribution. This change is associated with the precipitation of Zr-containing dispersoids and their role as nucleation sites for other precipitates. In the sample containing 0.15 wt.% Zr (Figure 3c), the fine precipitates appear more densely and uniformly distributed throughout the matrix. At this Zr content, the alloy exhibits one of the most homogeneous precipitate distributions compared with the AA7075 alloys containing 0.075 and 0.30 wt.% Zr. The fine, uniformly distributed particles are expected to increase hardness and strength by restricting dislocation motion. Increasing the Zr content to 0.30 wt.% (Figure 3d) results in more distinct grain boundaries and the presence of coarse or discontinuous secondary phases in some regions. At this higher Zr content, the concentration of particles at grain boundaries or in localized regions may be associated with the formation of coarse Zr-containing intermetallic phases due to the limited solubility of Zr. This suggests that the homogeneous distribution achieved at lower Zr contents is partially disrupted by the higher Zr addition.

When the SEM images and EDS results are evaluated together, the microstructure after RRA heat treatment is found to consist of an Al-rich α-Al matrix, pores, and localized secondary phases enriched in various alloying elements. The literature reports that secondary phases such as MgZn_2_ (η), Al_2_CuMg (S), Al_2_Cu (θ), and Al_7_Cu_2_Fe may commonly be present in AA7075 alloys [18,19].

In AA7075 alloy (Figure 3a), the matrix appears generally homogeneous, although irregular boundaries and a limited number of small pores are present. EDS analyses show that the Al content ranges from 95.26 to 97.57%, while the Mg and Si contents range from 1.71 to 3.25% and from 0.31 to 0.80%, respectively. These results indicate that the analyzed points represent the Al-based matrix and regions containing varying amounts of Mg and Si.

At some points, the Fe and Cu contents reached 0.87% and 0.71%, respectively. These results suggest the localized presence of Fe- and Cu-containing secondary phases within the matrix. However, the measured Zn content of 0–0.28% is lower than expected considering the nominal composition of AA7075. The Mg, Zn, and Cu contents detected by EDS suggest that secondary phases characteristic of AA7075, such as MgZn_2_, Al_2_CuMg, and Al_2_Cu, may be present within the matrix, while Al–Cu–Fe-based intermetallics may have formed in Fe-rich regions.

In the AA7075 alloy containing 0.075 wt.% Zr (Figure 3b), numerous small pores or particle-like features are observed within the matrix. Point EDS analyses show that the Zr content ranges from 0.19 to 1.76%. Although Zr was detected at every analyzed point, suggesting its distribution across the examined regions of the matrix, the variation in local concentrations indicates that the distribution is not fully homogeneous.

The Zr content exceeds 1% at points 1, 3, 4, and 6, indicating localized Zr-rich regions that may be associated with Al_3_Zr intermetallics. In addition, the Mg content reaches 8.21% and 7.57% at points 2 and 5, respectively, suggesting the presence of Mg-rich secondary phases, potentially MgZn_2_ or Al_2_CuMg.

In the AA7075 alloy containing 0.15 wt.% Zr (Figure 3c), fine features appear more densely and uniformly distributed within the matrix. The Zr content ranges from 0.82 to 2.02% at most analyzed points, whereas only 0.03% was measured at point 5. This variation suggests that Zr is not uniformly distributed solely in solid solution and that Zr-rich phases may have formed in certain regions.

**Figure 4:**
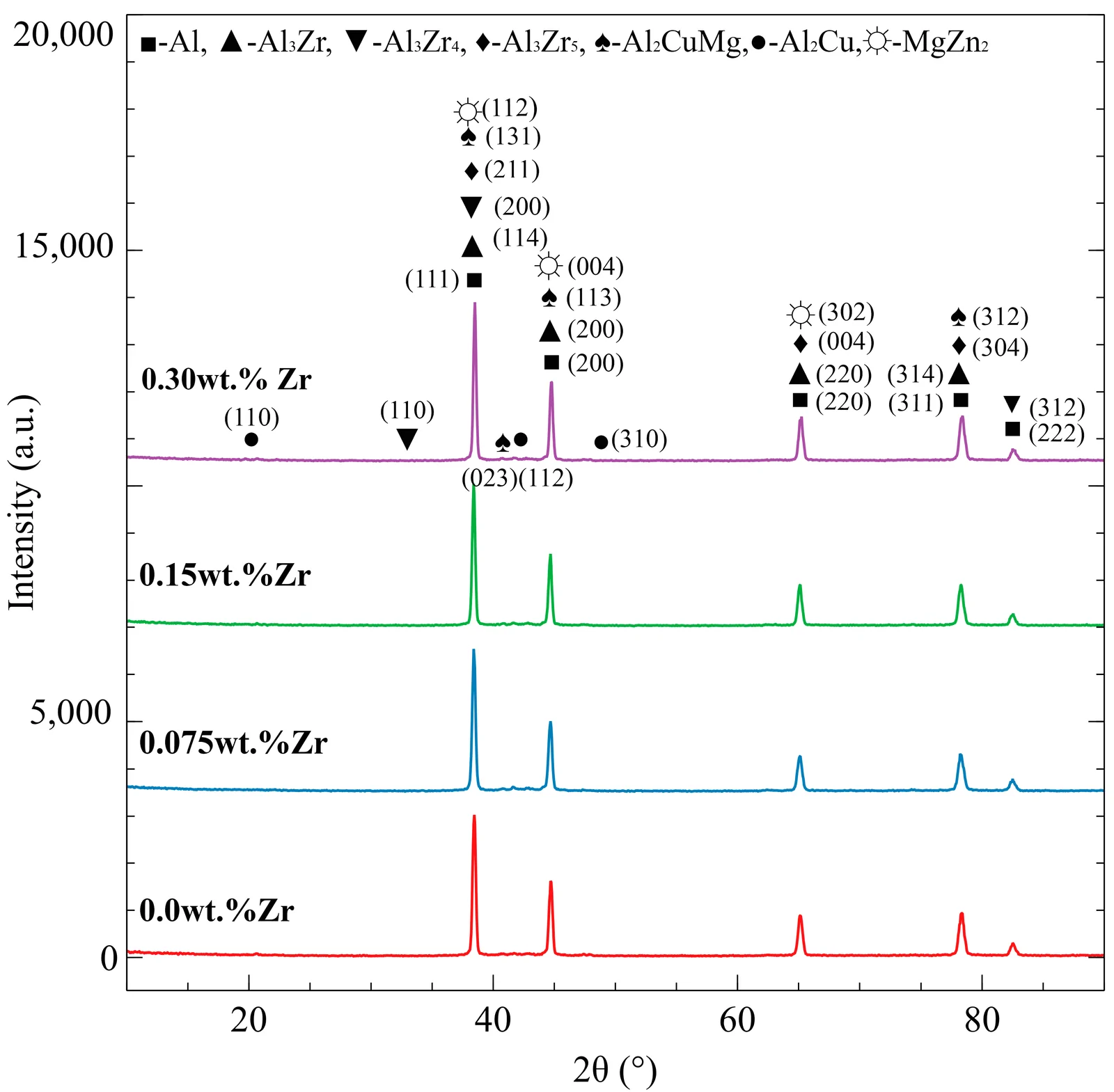
XRD analysis results for the AA7075 and AA7075 + Zr alloys.

In the AA7075 alloy containing 0.30 wt.% Zr (Figure 3d), localized coarse secondary phases are observed at grain boundaries compared with the other Zr-modified AA7075 alloys. The EDS results show Zr contents ranging from 0.34 to 2.25%. These regions are thought to contain Al_3_Zr, Al_3_Zr_4_, and Al_3_Zr_5_ intermetallic phases. The highest Zr contents were measured at points 3 and 7, reaching 2.18% and 2.25%, respectively, indicating more pronounced local Zr enrichment with increasing Zr addition. Furthermore, the Mg content reached 12.83% at point 2, indicating substantial Mg enrichment. Regions with high Mg contents may be associated with Mg-containing secondary phases such as MgZn_2_ or Al_2_CuMg. Figure 4 presents the XRD results for the AA7075 and AA7075 + Zr alloys after RRA heat treatment.

The XRD results suggested the possibility of Al_3_Zr, Al_3_Zr_4_, and Al_3_Zr_5_ phases in the Zr-containing AA7075 alloy. In particular, the low-intensity reflections at approximately 33°, 41–42°, and 82–83° in the sample containing 0.30 wt.% Zr may be indicative of the presence of Zr containing intermetallic phases. The weak intensities of the reflections may be attributable to the low nominal Zr content and the relatively small amounts of the relevant phases [20]. The phase assignments are, however, very tentative, and the presence of Al_3_Zr, Al_3_Zr_4_ and Al_3_Zr_5_ cannot be confirmed unambiguously from the XRD data presented here alone. This finding is consistent with the local Zr enrichment observed in the SEM images and the Zr contents of 2.18–2.25% measured at specific points by EDS. Accordingly, the local Zr contents exceeding the nominal composition in point EDS may be associated with Zr rich regions or intermetallic particles; however, EDS does not establish their specific phase identities. The XRD results and the Zr and Mg rich regions observed by SEM–EDS may be associated with secondary-phase formation. The high-intensity peaks observed at approximately 38°, 45°, 65°, 78°, and 82° in the XRD patterns of all samples correspond to the face-centered cubic α-Al matrix.

The XRD analysis also identified Al_2_CuMg, Al_2_Cu, and MgZn_2_ phases. The presence of Al_2_CuMg and Al_2_Cu is consistent with the detection of Cu in certain regions by EDS. Similarly, the identification of MgZn_2_ indicates the formation of Mg- and Zn-containing η/η′-type precipitates during RRA heat treatment. The Mg contents of 7.57–12.83% measured at some EDS analysis points also support the presence of Mg-rich precipitates or segregated regions. However, the MgZn_2_ precipitates could not be individually identified by EDS because of their small size.

The SEM images presented in Figure 3 show that, after RRA heat treatment, the AA7075 alloy consists of an Al-rich matrix, pores, and secondary-phase regions enriched in various alloying elements. The XRD patterns of all samples are dominated by the main α-Al peaks. Furthermore, the identification of peaks corresponding to Al_3_Zr, Al_3_Zr_4_, and Al_3_Zr_5_ intermetallic phases in the Zr-containing samples indicates that some of the Zr forms Zr-rich secondary phases within the Al matrix. The Zr content of up to 2.25% measured by EDS further supports the presence of these phases. The pronounced Zr enrichment and coarser secondary-phase regions, particularly in the sample containing 0.30 wt.% Zr, suggest increased intermetallic phase formation with increasing Zr content. The Al_2_CuMg, Al_2_Cu, and MgZn_2_ phases identified by XRD indicate the formation of Cu-, Mg-, and Zn-containing precipitates during RRA heat treatment.

The more homogeneous distribution of fine features in the samples containing 0.075 and 0.15 wt.% Zr may be attributed to Zr-containing dispersoids promoting precipitate nucleation and inhibiting grain/particle boundary migration. Increasing the Zr content to 0.30 wt.% leads to localized Zr enrichment and the formation of coarser intermetallic phases, indicating a partial loss of microstructural homogeneity at the higher Zr content.

### 3.2. Microhardness and Density Results of AA7075 and AA7075 + Zr Alloys

**Figure 5:**
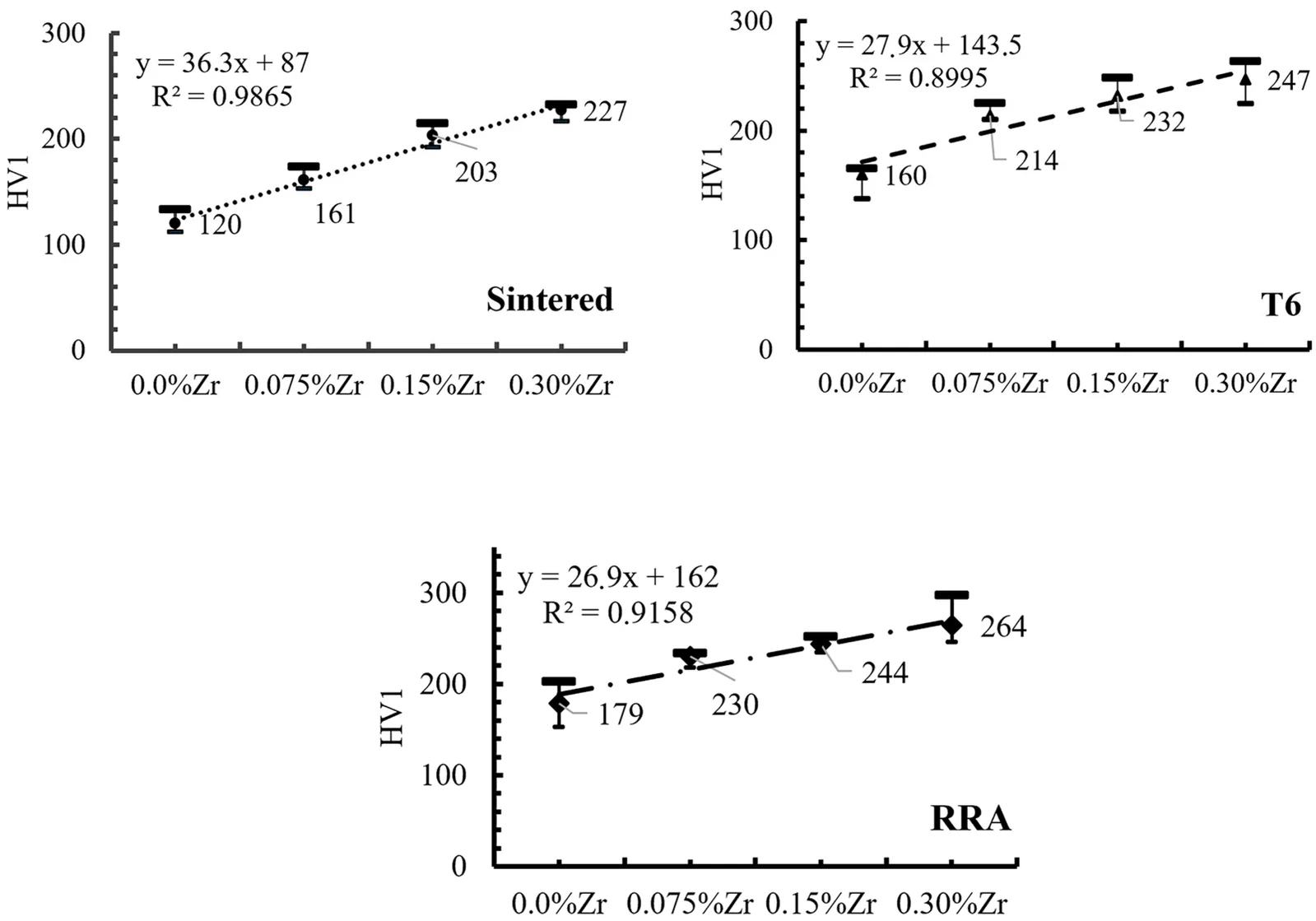
Variation in microhardness as a function of Zr content (wt.%).

**Figure 6:**
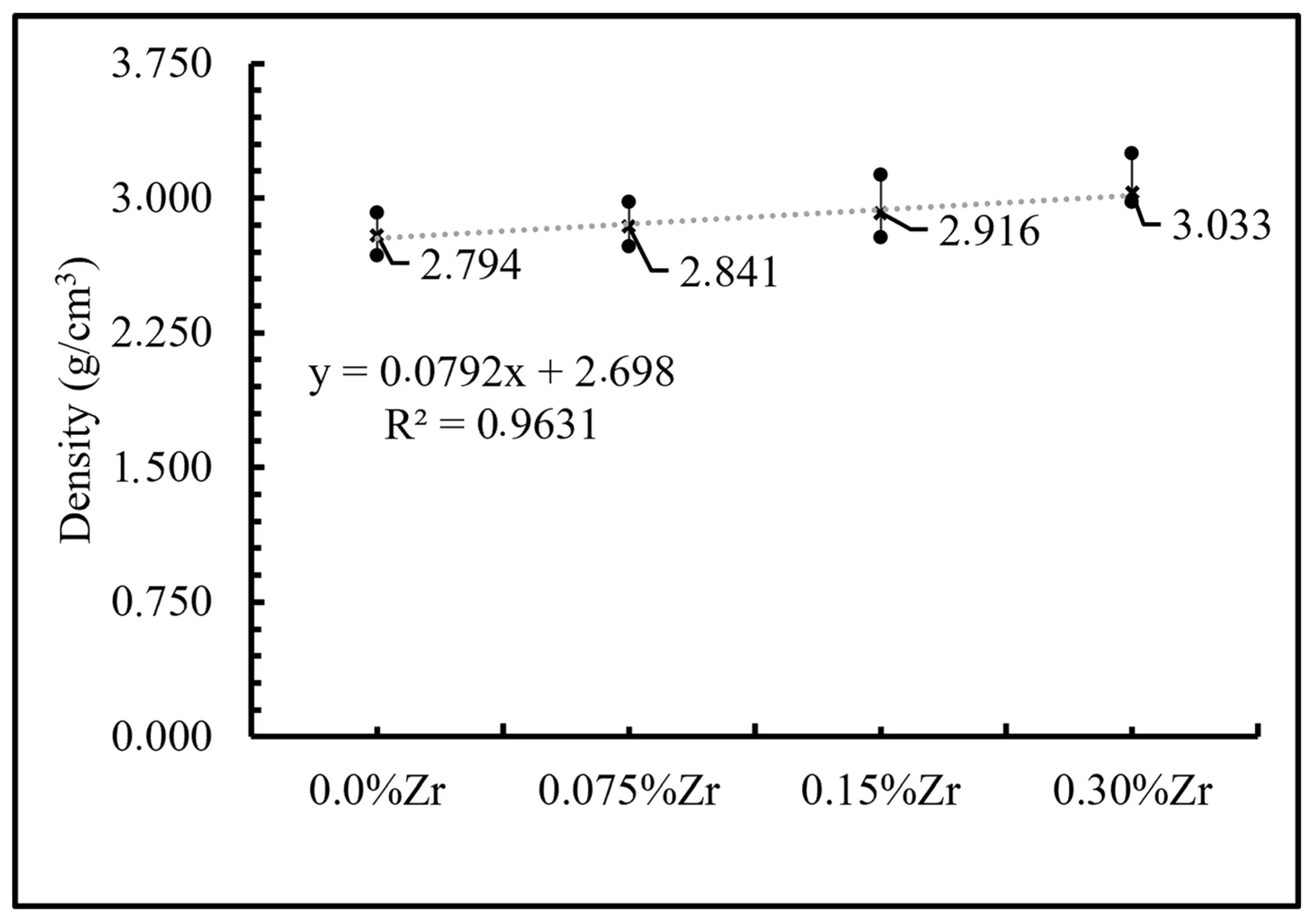
Density of the AA7075 and AA7075 + Zr alloys (wt.%).

The microhardness results and density measurements of the AA7075 and AA7075 + Zr alloys after RRA heat treatment are presented in Figure 5 and Figure 6, respectively.

As shown in Figure 5, increasing the Zr content raised the microhardness of the alloys from 120 to 227 HV1 after sintering, from 160 to 247 HV1 after T6 heat treatment, and from 179 to 264 HV1 after RRA heat treatment. The highest value was measured in the RRA-treated sample containing 0.30 wt.% Zr and was approximately 47.5% higher than that of the AA7075 sample subjected to the same treatment. This increase is associated with the restriction of dislocation motion by MgZn_2_, Al_2_CuMg, and Al_2_Cu precipitates formed during RRA treatment, together with Al–Zr-based dispersoids and intermetallic phases formed through Zr addition [21,22,23,24]. The Zr-rich regions identified in the XRD and EDS results presented in Figure 4 and Table 2 further support this interpretation. The increase in microhardness observed with Zr addition is consistent with the findings reported by Kumar et al. [25] for Al7075. The authors reported that the microhardness of samples produced by laser-directed energy deposition (LDED) increased from 80.1 to 85.7 HV as the Zr content increased from 0.5 to 1 wt.% but decreased to 70.6 HV at 1.5 wt.% Zr. 

In a study on AA7075 alloy produced by PM, Şimşek attributed the increase in hardness after aging to the formation of MgZn_2_ precipitates that hinder dislocation motion. When interpreted in terms of the secondary phases and precipitate structure, the reported hardness results are consistent with those of the present study. Aging heat treatment increased the hardness of the AA7075 alloys [26].

The experimental density of the AA7075 alloy was measured as 2.794 g/cm^3^. With additions of 0.075, 0.15, and 0.30 wt.% Zr, the density increased to 2.841, 2.916, and 3.033 g/cm^3^, respectively. As shown in Figure 6, these values correspond to increases of approximately 1.7%, 4.4%, and 8.6%, respectively. In materials produced by powder metallurgy, an increase in measured density is generally associated with reduced porosity, improved interparticle contact, and the formation of stronger bonds during sintering [2,27].

### 3.3. Surface Roughness Results of AA7075 and AA7075 + Zr Alloys

Following RRA heat treatment, the surface roughness values (Ra) of the AA7075 and AA7075 + Zr alloys were measured pre-corrosion and post-corrosion tests. Five surface roughness measurements were performed for each alloy, and the mean values were calculated.

In pre-corrosion testing, the highest surface roughness was measured in the AA7075 alloy (2.054 µm), while the lowest was recorded in the AA7075 + 0.30 wt.% Zr alloy (0.078 µm) (Table 3). The initial surface roughness decreased with increasing Zr content. After corrosion testing, the surface roughness increased in all AA7075 and AA7075 + Zr alloys. The highest value was measured in the AA7075 alloy (2.684 µm), whereas the lowest was observed in the AA7075 + 0.30 wt.% Zr alloy (0.102 µm). However, the largest increase in surface roughness pre- and post-corrosion testing was recorded in the AA7075 + 0.075 wt.% Zr alloy (+0.717 µm). 

Mopon et al. [28] investigated the corrosion behavior of AA7075-T6 in a NaCl environment. They reported dissolution of intermetallic particles and the surrounding Al matrix in the NaCl solution. Such dissolution and degradation can create height variations on the sample surfaces.

### 3.4. Corrosion Results of AA7075 and AA7075 + Zr Alloys

**Figure 7:**
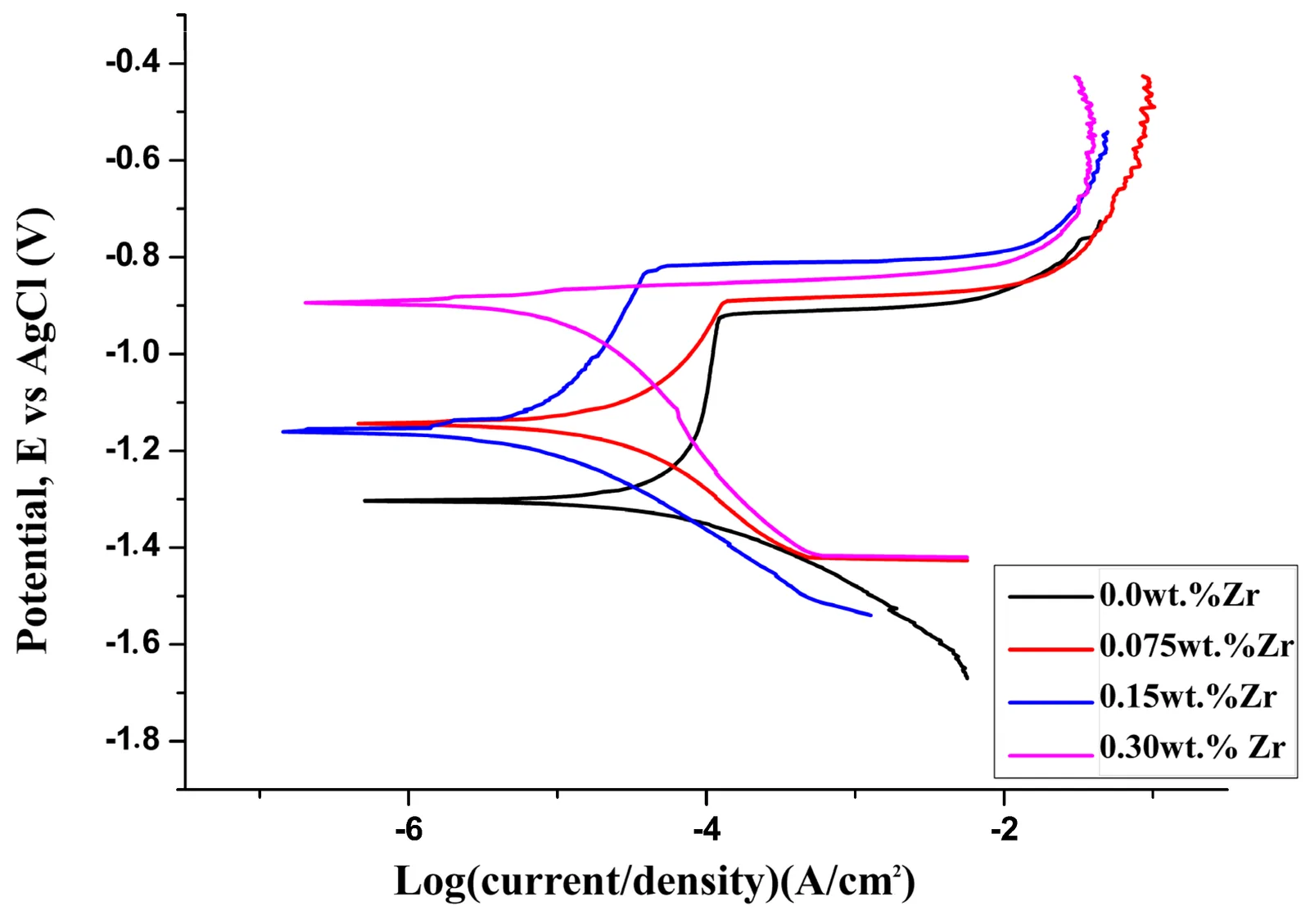
Potentiodynamic polarization curves of AA7075 and AA7075 + Zr alloys in 3.5% NaCl solution.

To determine the effect of Zr content on corrosion behavior, the RRA-treated AA7075 and AA7075 + Zr alloys containing different amounts of Zr were immersed in a solution containing 3.5% NaCl for 3600 s to allow the open-circuit potential (OCP) to stabilize. The potentiodynamic polarization curves are presented in Figure 7. The corrosion potential (E_corr_), corrosion current density (j_corr_) and corrosion rate values obtained from these curves are reported in Table 5.

**Table 4:** Corrosion parameters of AA7075 and AA7075 + Zr alloys.

Solution	Alloys	$E_{corr}$ (V)	$j_{corr}$	*I_corr_* (μA)	Corrosion Rate (mm/year)	Open Circuit Potential OCP (V)	E_pitt_
3.5% NaCl	0.0 wt.% Zr	−1.32	151.4	16.2	1.9	−0.96726	−0.92
	0.075 wt.% Zr	−1.15	31.7	3.4	0.39	−0.98542	−0.89
	0.15 wt.% Zr	−1.14	19.63	2.1	0.23	−0.99471	−0.82
	0.30 wt.% Zr	−0.89	15.88	1.7	0.19	−1.00012	−0.85

Figure 7 presents the potentiodynamic polarization curves of the AA7075 and AA7075 + Zr alloys. Although the curves exhibit broadly similar behavior, Zr addition produced marked changes in the corrosion parameters. As shown in Table 4, the highest E_corr_ and the lowest corrosion j_corr_ were obtained for the alloy containing 0.30 wt.% Zr. The lower j_corr_ indicates a reduced metal dissolution rate and improved corrosion resistance. The shift in E_corr_ toward more positive values suggests more noble electrochemical behavior [29].

As the Zr content increased, the corrosion potential shifted toward more positive values, while the corrosion current density and corrosion rate decreased. These results indicate that Zr addition improved the corrosion resistance of AA7075, with the highest resistance achieved in the alloy containing 0.30 wt.% Zr. The E_corr_ value increased from −1.32 V for the AA7075 alloy to −0.89 V with the addition of 0.30 wt.% Zr. The pitting potential (E_pitt_) increased from −0.91 V for the AA7075 alloy to −0.85 V for the alloy with 0.30 wt.% Zr. The 70 mV positive shift indicates that a higher potential is required to initiate pitting and thus a greater pitting resistance has been achieved under the conditions of this study. Meanwhile, j_corr_ and the corrosion rate decreased consistently with increasing Zr content. The lowest corrosion rate was measured in the alloy containing 0.30 wt.% Zr, whereas the highest was recorded in the AA7075 alloy. These findings demonstrate that Zr addition improved the corrosion resistance of AA7075 in a 3.5% NaCl environment, with the most pronounced improvement obtained at 0.30 wt.% Zr. The stabilized open-circuit potential (OCP) and E_corr_ values are presented in Table 4. The stabilized OCP value was −0.967 V for the AA7075 alloy, whereas it shifted to −1.000 V for the alloy containing 0.30 wt.% Zr. In contrast, E_corr_ has shifted from −1.32 V to −0.89 V. Consequently, the OCP and E_corr_ values exhibited different trends as the Zr content increased. It is considered that these differing trends may be related to surface conditions which are altered by the addition of Zr and to the electrochemical processes that occur during polarization. However, since a more negative OCP value does not, by itself, imply a higher corrosion rate, OCP values should be evaluated in conjunction with corrosion current density. In this context, the decrease in corrosion current density with increasing Zr content indicates improved corrosion behavior under the experimental conditions used.

When the obtained corrosion current densities are compared with the values reported in the literature for the AA7075 alloy, it is observed that the values in this study are higher. It has been reported in previous studies that additives can influence the corrosion behavior of AA7075 alloys produced from powder. Ebina et al. [30] reported that, in samples produced from AA7075 and Mn powders via spark plasma sintering, the addition of Mn improved corrosion behavior in a 0.1 M NaCl solution. Arsun et al. [31] reported the positive effect of adding hydrothermal carbon on the corrosion properties of AA7075-based composites produced via powder metallurgy. In this study, a decrease in corrosion current density was also observed with the addition of Zr. However, it is considered that differences in the additives used, production methods, and experimental conditions in the studies may influence the corrosion results obtained. When the obtained corrosion current densities are compared with the values reported in the literature for the AA7075 alloy, it is observed that the values in this study are higher. In a previous study, the corrosion current density of the AA7075 alloy in a 3.5% NaCl solution was reported to be 1.60 µA/cm^2^ [32]. In the present work, the corrosion current density was determined to be 151.4 µA/cm^2^ for the AA7075, while it was found to be 15.88 µA/cm^2^ for the sample containing 0.30wt.% Zr. Differences between studies may be related to the alloy’s production method, microstructure, surface condition, heat treatment conditions, and electrochemical experimental parameters. Furthermore, the decrease in corrosion current density observed with the addition of Zr in the present study indicates an improvement in corrosion behavior among the samples examined.

**Figure 8:**
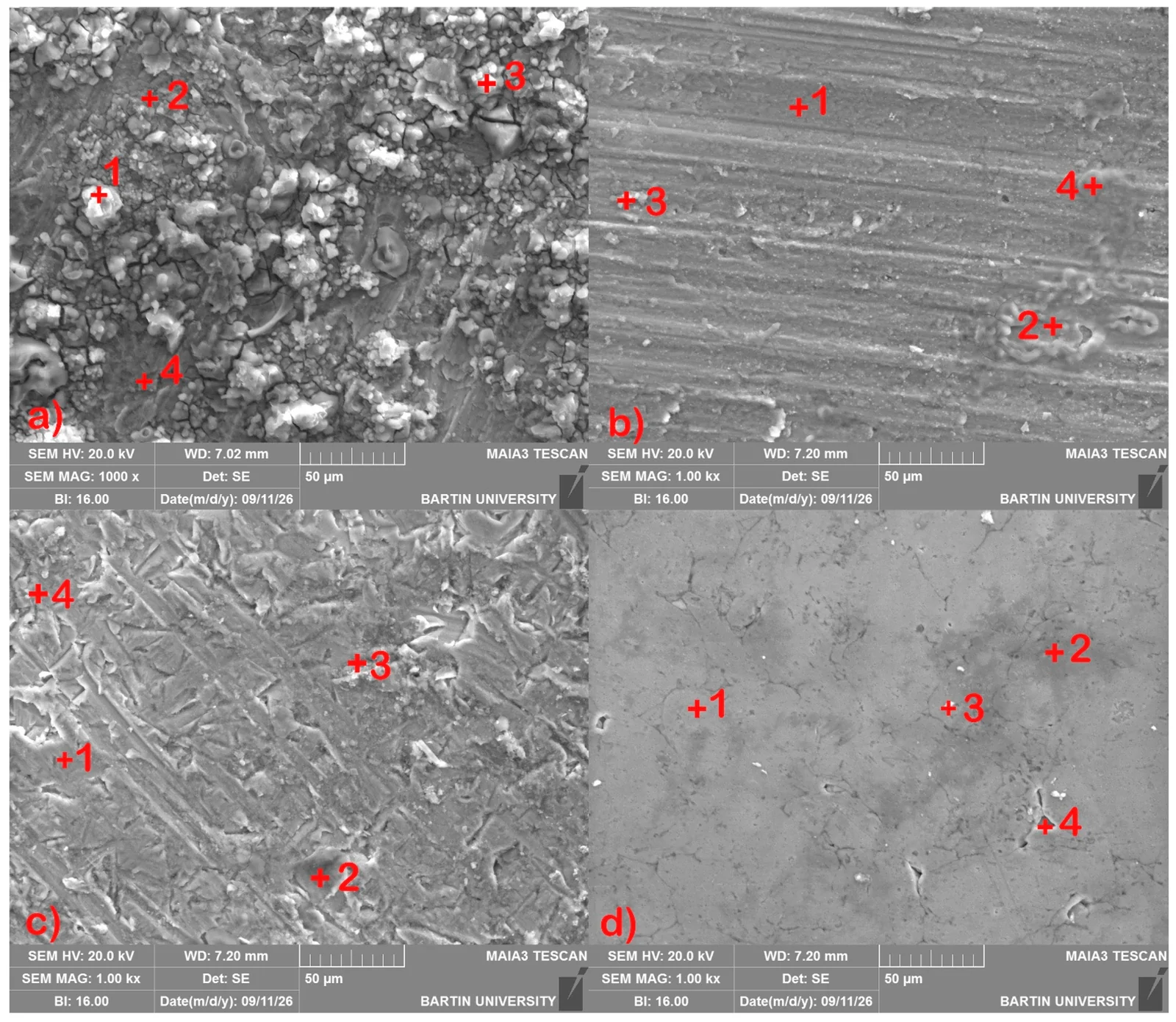
SEM images of the post-corrosion surfaces of AA7075 alloys containing (**a**) 0.0 wt.% Zr, (**b**) 0.075 wt.% Zr, (**c**) 0.15 wt.% Zr, and (**d**) 0.30 wt.% Zr.

The results obtained from the potentiodynamic polarization curves are consistent with the electrochemical parameters presented in Table 4. Figure 8 presents SEM images of the surfaces of the AA7075 and AA7075 + Zr alloys post-corrosion testing.

The post-corrosion SEM images reveal severe corrosion-induced degradation on the surface of the AA7075 alloy. Figure 8a shows a dense corrosion layer and corrosion pits on the surface of the AA7075 alloy. Considering Figure 8a and the EDS results in Table 5, O contents of 65.3% and 60.9% were detected in regions 1 and 3, respectively, suggesting the presence of Al-based hydroxide and oxide corrosion layers in these regions.

**Table 5:** Post-corrosion EDS analysis results for the AA7075 and AA7075 + Zr alloys.

Alloys	Location	Mg	Al	Si	Mn	Fe	Cu	Zn	Zr	Na	Cl	O
0.0 wt.% Zr	1	0.80	30.3	0.00	0.00	0.00	0.00	1.20	-	1.70	0.50	65.30
	2	2.30	62.0	0.10	0.10	0.30	2.30	3.80	-	0.90	0.20	28.00
	3	0.60	33.8	0.00	0.00	0.10	0.10	2.10	-	1.50	0.80	60.90
	4	3.20	72.6	0.20	0.10	0.30	1.80	4.30	-	0.80	0.10	16.60
0.075 wt.% Zr	1	2.10	68.8	0.10	0.10	0.30	1.30	3.60	0.10	0.60	0.00	23.00
	2	1.50	39.8	0.00	0.10	0.20	1.00	1.80	0.00	1.00	0.20	54.50
	3	1.80	55.1	0.70	0.30	0.90	2.20	1.80	0.10	0.60	0.10	36.40
	4	0.80	34.3	0.00	0.10	0.20	0.80	0.70	0.00	1.50	0.10	61.50
0.15 wt.% Zr	1	2.00	80.9	0.00	0.10	0.30	1.60	3.90	0.10	0.80	0.00	10.20
	2	1.60	64.3	0.60	0.30	0.70	2.90	3.50	0.30	4.50	0.30	21.30
	3	1.10	25.2	24.6	0.10	0.60	0.40	0.90	0.20	1.90	0.10	45.00
	4	1.80	58.1	13.2	0.10	0.40	1.20	3.30	0.20	2.00	0.10	19.70
0.30 wt.% Zr	1	1.40	87.5	0.00	0.10	0.10	1.40	4.80	0.40	0.40	0.10	3.90
	2	2.10	75.5	0.10	0.10	0.10	1.20	4.00	0.30	0.80	0.10	15.70
	3	5.80	52.8	0.00	0.10	0.30	0.60	1.70	0.00	0.50	0.10	38.00
	4	2.30	77.0	0.00	0.20	0.10	4.40	11.10	0.50	0.40	0.20	3.80

Furthermore, the increase in Al content to 72.6% and the decrease in O content to 16.6% in region 4 indicate a thinner corrosion layer at this location. In chloride-containing environments, Cl^−^ ions can locally disrupt the naturally occurring passive oxide/hydroxide film on the aluminum surface, initiating pitting corrosion [29]. Following this local breakdown, anodic dissolution and the accompanying hydrolysis reactions can lead to the formation of aluminum oxide-based corrosion products containing Al^3+^ centers and hydroxyl groups [33]. Therefore, based on the present EDS findings, the structures formed on the surface may be interpreted as Al-based oxide/hydroxide corrosion products.

On the surface of the AA7075 sample containing 0.075 wt.% Zr (Figure 8b), the O contents in regions 2–4 range from 54.5 to 61.5% (Table 5), suggesting the formation of an oxygen-rich corroded surface in these regions. In particular, region 4 contains 61.5% O and 34.3% Al, indicating a surface rich in corrosion products. Thus, the addition of 0.075 wt.% Zr appears to partially improve the surface morphology but is insufficient to form a homogeneous and stable protective layer.

The surface of the AA7075 sample containing 0.15 wt.% Zr (Figure 8c) exhibits fewer corrosion products than those of the AA7075 sample and the sample containing 0.075 wt.% Zr. The detection of 80.9% Al and only 10.2% O in region 1 (Table 5) suggests less extensive corrosion on the surface of this sample.

The AA7075 containing 0.30 wt.% Zr exhibited the smoothest and least degraded surface morphology (Figure 8d). The low O contents of only 3.9% and 3.8% in regions 1 and 4, respectively (Table 5), indicate that the EDS interaction volume in these regions predominantly represents the metallic matrix. In contrast, the O contents of 15.7% in region 2 and 38.0% in region 3 suggest that corrosion was not completely eliminated but remained confined to more limited, localized areas. In particular, 11.1% Zn, 4.4% Cu, and 2.3% Mg were detected in region 4 of the AA7075 sample containing 0.30 wt.% Zr. This composition suggests a Zn–Mg–Cu-rich precipitate or intermetallic region remaining post-corrosion.

As the Zr content increases, the dense corrosion layer on the sample surfaces becomes less extensive. The average O content calculated from the EDS results decreases from 42.7% in the AA7075 alloy to 14.6% in the alloy with the highest Zr content. These findings suggest that corrosion damage decreases with increasing Zr content. The effect of Zr may also be associated with fine, thermally stable Al_3_Zr dispersoids that can form during heat treatment.

When the post-corrosion SEM images and EDS results presented in Figure 8 and Table 5 are evaluated together, dissolution and pitting damage are observed on the sample surfaces. Although the passive oxide film that naturally forms on AA7075 provides some protection against general corrosion, Cl^−^ ions in the NaCl solution can disrupt this film at weak regions and initiate pitting corrosion. Ładak et al. [34] observed crystallographic dissolution and localized corrosion damage in AW7075 tested in a 3.5% NaCl solution, depending on the heat treatment condition. They reported that the RRA-treated sample exhibited a lower general corrosion rate than the T6-treated and two-stage-aged samples.

In the 3.5% NaCl solution, the expected anodic process is the dissolution of the Al matrix, which may be represented by the following simplified reaction:

Al → Al^3+^ + 3e^−^ (2)

Since the solution was not deaerated, dissolved oxygen reduction is expected to contribute to the cathodic process [35]:

O_2_ + 2H_2_O + 4e^−^ → 4OH^−^ (3)

These equations describe the expected reactions under experimental conditions. Oxygen reduction is not necessarily the only cathodic process, as hydrogen evolution may also be occurring to some extent depending on the local conditions and the applied potential [36].

**Figure 9:**
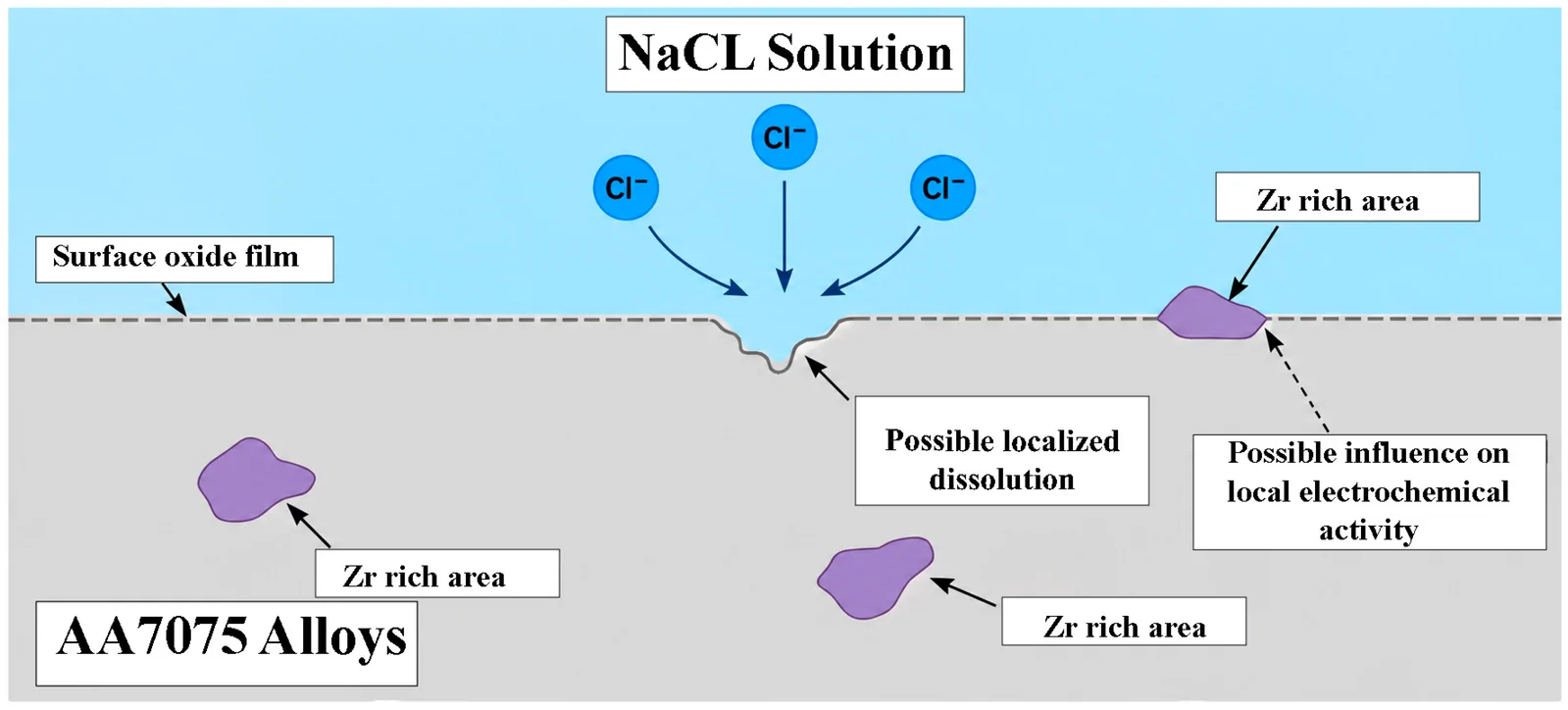
Schematic representation of the proposed corrosion processes and the potential role of Zr-rich regions in AA7075 + Zr alloys exposed to a NaCl solution.

The local corrosion mechanism as inferred from the corrosion data and microstructural observations is illustrated schematically in Figure 9. The mechanism is thought to involve chloride ions promoting local breakdown of the surface oxide film and hence local dissolution (pitting corrosion) within the AA7075 matrix. It is also proposed that the Zr-rich regions may affect the local electrochemical behavior and, therefore, the initiation and progression of corrosion.

This behavior has been attributed to RRA-induced changes in the size and continuity of grain boundary precipitates, which limit preferential anodic dissolution pathways along grain boundaries. In the present study, the reduction in corrosion damage with increasing Zr content may be associated with the effects of Zr on the grain structure and precipitate distribution, in addition to the precipitate modifications induced by RRA treatment.

## 4. Conclusions

In this study, different amounts of Zr were added to AA7075, and the alloys were subjected to RRA heat treatment. The findings from the characterization and corrosion testing of the AA7075 and AA7075 + Zr alloys are summarized below.

- XRD analysis identified Al, Al_2_CuMg, Al_2_Cu, and MgZn_2_ phases, as well as the Zr-containing intermetallic phases Al_3_Zr, Al_3_Zr_4_, and Al_3_Zr_5_.
- With increasing Zr content, the microhardness increased from 179 HV1 in AA7075 + 0.0 wt.% Zr to 264 HV1 in AA7075 + 0.30 wt.% Zr, corresponding to an increase of approximately 47.5%.
- Zr addition increased the density from 2.794 g/cm^3^ in AA7075 + 0.0 wt.% Zr to 3.033 g/cm^3^ in AA7075 + 0.30 wt.% Zr, corresponding to an increase of approximately 8.6%.
- Pre-corrosion testing, surface roughness decreased with increasing Zr content, from 2.054 µm in AA7075 + 0.0 wt.% Zr to 0.078 µm in AA7075 + 0.30 wt.% Zr. Surface roughness increased post-corrosion testing. The largest increase was measured in AA7075 + 0.075 wt.% Zr (+0.717 µm), while the smallest occurred in AA7075 + 0.30 wt.% Zr (+0.024 µm). The alloy containing 0.30 wt.% Zr exhibited the lowest surface roughness both pre- and post-corrosion testing.
- In the 3.5% NaCl environment, the corrosion rate decreased from 1.90 mm/year in AA7075 + 0.0 wt.% Zr to 0.19 mm/year in AA7075 + 0.30 wt.% Zr, corresponding to a reduction of approximately 90%.
- Post-corrosion SEM images showed surface features consistent with pitting corrosion.
- Overall, Zr addition improved the microhardness and corrosion properties of RRA-treated AA7075.

## Figures and Tables

**Table 1:** Chemical compositions of the AA7075 and Zr powders used in this study (wt.%).

Materials	Zn	Mg	Cu	Fe	Si	Zr	Mn	Cr	Al	Zr
AA7075	5.9	2.7	1.8	0.4	0.3	0.03	0.15	0.18	Balance	-
Zr										99.99

**Table 2:** EDS analysis results for the AA7075 and AA7075 + Zr alloys.

Alloys	Location	Mg	Al	Si	Mn	Fe	Cu	Zn	Zr
0.0 wt.% Zr	1	1.71	96.70	0.31	0.12	0.87	0.00	0.28	-
	2	3.25	95.57	0.55	0.00	0.00	0.60	0.03	-
	3	2.90	95.93	0.57	0.23	0.03	0.27	0.07	-
	4	2.79	95.26	0.80	0.00	0.45	0.71	0.00	-
	5	1.97	97.57	0.46	0.00	0.00	0.00	0.00	-
0.075 wt.% Zr	1	3.27	94.29	0.60	0.04	0.00	0.41	0.15	1.25
	2	8.21	91.06	0.54	0.00	0.00	0.00	0.00	0.19
	3	3.34	94.23	0.57	0.00	0.19	0.48	0.00	1.18
	4	3.49	93.29	0.89	0.00	0.47	0.00	0.09	1.76
	5	7.57	89.93	0.74	0.11	0.01	0.65	0.00	0.99
	6	3.63	94.23	0.40	0.00	0.45	0.00	0.04	1.24
0.15 wt.% Zr	1	2.01	95.07	0.62	0.15	0.00	0.24	1.03	0.87
	2	2.76	94.09	0.48	0.40	0.00	0.68	0.34	1.24
	3	5.38	90.00	1.22	0.49	0.09	0.38	0.41	2.02
	4	3.66	97.23	0.73	0.10	0.34	0.11	0.00	0.82
	5	2.80	97.04	0.11	0.00	0.01	0.00	0.00	0.03
	6	2.41	94.77	0.88	0.00	0.00	0.00	0.00	1.93
	7	1.08	9.27	89.47	0.18	0.00	0.00	0.00	0.00
0.30 wt.% Zr	1	2.36	96.50	0.37	0.12	0.00	0.00	0.12	0.53
	2	12.83	84.82	0.54	0.34	0.00	0.48	0.65	0.34
	3	4.11	92.44	0.62	0.08	0.00	0.57	0.00	2.18
	4	3.47	94.61	0.54	0.28	0.16	0.14	0.00	0.81
	5	3.82	94.12	0.56	0.36	0.24	0.00	0.25	0.66
	6	4.15	93.83	0.50	0.10	0.07	0.17	0.00	1.17
	7	2.45	94.80	0.45	0.00	0.00	0.00	0.05	2.25

**Table 3:** Surface roughness results for the AA7075 and AA7075 + Zr alloys.

Alloys	Surface Roughness Ra (µm)		
	Pre-Corrosion Test	Post-Corrosion Test	ΔRa (µm)
0.0 wt.% Zr	2.054	2.684	+0.630
0.075 wt.% Zr	1.142	1.859	+0.717
0.15 wt.% Zr	0.947	1.394	+0.447
0.30 wt.% Zr	0.078	0.102	+0.024

## Data Availability

The original contributions presented in this study are included in the article. Further inquiries can be directed to the corresponding author.
